# Tumors on the Shoulder1Read before the semi-annual meeting of the Pennsylvania State Veterinary Medical Association, Erie, Pa., September, 1900.

**Published:** 1900-12

**Authors:** W. S. Phillips

**Affiliations:** Reading, Pa.


### TUMORS ON THE SHOULDER.[1]

BY W. S. PHILLIPS, V.S.,

READING, PA.

TUMORS on the shoulder consist of a hardened swelling in the levator humeri and the pectoral muscle, which soon takes on unhealthy adhesions, and in some cases are as large as six or eight inches in circumference. Met with in carriage-horses, but mostly in draught-horses; generally caused by ill-fitting collars, causing unequal pressure. Have had considerable trouble with six or eight cases recently, the majority of which had been treated. Found the parts very much swollen and false adhesions formed. First paid attention to the horse's diet, and for several days applied cooling lotions to reduce the swelling.

After poulticing, blistering and setoning, I find in the majority of cases the only way is to dissect out the whole enlargement, which sometimes penetrates to the depth of five or six inches, appearing dangerous while operating. The wound is sometimes brought together above by several stitches, leaving the lower part of the wound open, aiding the discharge to escape. Now I apply cooling lotions externally once a day, and syringe the wound once daily with a lotion of sulphate of zinc, which I find the best. The wound heals in a short time.

[1] Read before the semi-annual meeting of the Pennsylvania State Veterinary Medical Association, Erie, Pa., September, 1900.

I met with a case about eight weeks ago. The subject was a white mule. There were two enlargements, inflamed and hardened. Took into consideration melanosis, as this appeared a suspicious case, being a white mule. First used lotions, then a stimulating liniment, which contained a few ounces of liquid blister (used this daily) for several weeks, which did not have much vesicating effect. The patient was about six miles from the city, and as the liniment had all been used the owner then poulticed, and in the course of a week brought the patient in. I found the smallest tumor had disappeared. The larger one soft and fluctuating. I lanced it and found it contained six ounces of healthy pus. Made a free opening, using the sulphate of zinc solution as before; also cooling applications externally once daily.

